# Development and Validity of the Rating-of-Fatigue Scale

**DOI:** 10.1007/s40279-017-0711-5

**Published:** 2017-03-10

**Authors:** D. Micklewright, A. St Clair Gibson, V. Gladwell, A. Al Salman

**Affiliations:** 1 School of Sport, Rehabilitation and Exercise Sciences, University of Essex, Wivenhoe Park, Colchester, Essex, CO4 3SQ UK; 2 Faculty of Health, Sport and Performance, University of Waikato, Hamilton, New Zealand; 3 University of Dammam, Dammam, Kingdom of Saudi Arabia

## Abstract

**Objective:**

The purpose of these experiments was to develop a rating-of-fatigue (ROF) scale capable of tracking the intensity of perceived fatigue in a variety of contexts.

**Methods:**

Four experiments were carried out. The first provided the evidential basis for the construction of the ROF scale. The second tested the face validity of the ROF, and the third tested the convergent and divergent validity of the ROF scale during ramped cycling to exhaustion and 30 min of resting recovery. The final experiment tested the convergent validity of the ROF scale with time of day and physical activity (accelerometer counts) across a whole week.

**Results:**

Modal selections of descriptions and diagrams at different levels of exertion and recovery were found during Experiment 1 upon which the ROF scale was constructed and finalised. In Experiment 2, a high level of face validity was indicated, in that ROF was reported to represent fatigue rather than exertion. Descriptor and diagrammatic elements of ROF reportedly added to the coherence and ease of use of the scale. In Experiment 3, high convergence between ROF and various physiological measures were found during exercise and recovery (heart rate, blood lactate concentration, oxygen uptake, carbon dioxide production, respiratory exchange ratio and ventilation rate were all *P* < 0.001). During ramped cycling to exhaustion ROF and RPE did correspond (*P* < 0.0001) but not during recovery, demonstrating discriminant validity. Experiment 4 found ROF to correspond with waking time during each day (Mon–Sun all *P* < 0.0001) and with physical activity (accelerometer count) (Mon–Sun all *P* < 0.001).

**Conclusions:**

The ROF scale has good face validity and high levels of convergent validity during ramped cycling to exhaustion, resting recovery and daily living activities. The ROF scale has both theoretical and applied potential in understanding changes in fatigue in a variety of contexts.

**Electronic supplementary material:**

The online version of this article (doi:10.1007/s40279-017-0711-5) contains supplementary material, which is available to authorized users.

## Key Points

**Table:** 

A new method of measuring perceived fatigue named the rating-of-fatigue (ROF) scale has been developed.
The ROF scale was found to have good face validity and high levels of convergent validity during ramped cycling to exhaustion exercise, resting recovery and daily living activities. The ROF scale was also found to discriminate between perceived exertion during recovery from exercise.
The intensity-based approach to measuring perceived fatigue adopted with the ROF scale appears to support theoretical notions that perceived fatigue should be regarded as a global perceptual phenomenon.

## Introduction

Fatigue has proven to be a nebulous concept and a challenging topic of research and, as such, has led to questions [1] about whether it will ever be possible to develop a global theory about its causes, mechanisms, consequences, prevention and treatment. The problem, as described in numerous recent publications, is in part due to the inherent difficulties of agreeing on a common definition of fatigue [1–5]. Fatigue has been described as a ubiquitous [6], multifactorial [1] and complex phenomenon [7] that must be studied from a holistic perspective [4]. Attempts to define and understand fatigue have either focused on or led to the emergence of fatigue dichotomies, the most common ones being central-peripheral [8, 9], physical-mental [10, 11], acute-chronic [12] and normal-pathological [13]. These dichotomies, while important to understanding and managing fatigue in particular applied contexts, nebulise rather than consolidate our theoretical understanding of fatigue [3]. Furthermore, they do very little to clarify the distinction between fatigue and other related concepts such as sleepiness [7], exertion [14, 15], effort [16–19], exhaustion [20] and malaise [21], a situation that is exacerbated by the inclusion of such adjectives in many fatigue scales where they are often used synonymously and without being operationally defined.

Recently, a useful distinction has been drawn between fatigue, described as a subjective sensation, and fatigability, described as objective change in motor or physical performance [3, 22]. While the authors acknowledge that perceptions of fatigue and fatigability have the potential to influence each other, they and others also cite instances where fatigue and fatigability are independent [16, 17, 23, 24]. Acknowledging the interactive psychophysiological nature of fatigue, Enoka and Deuchateau [25] have perhaps provided the most useful definition of fatigue to date which they describe as “…a disabling symptom in which physical and cognitive function is limited by interactions between performance fatigability and perceived fatigability.” In this definition they describe fatigue as a disabling symptom but crucially, rather than specify the direction of causality, they simply acknowledge that an interaction exists between perceptual and physiological fatigability. This is an important conceptual step because it gives rise to the possibility that fatigue and fatigability can act both dependently and independently of each other in ways that cause a variety of fatiguing effects.

Most previous approaches to measuring perceptions of fatigue have tended to use instruments designed for specific populations such as cancer patients [26–28] and multiple-sclerosis patients [29–31] that often include items that are specific to the signs or symptoms of a disease. Collectively, but unintentionally, this has reinforced the ubiquitous presence of fatigue yet created an impediment to inter-pathological comparisons of fatigue and the development of a generalised theory of fatigue. Most fatigue scales also comprise multiple items [26–34] that, owing to their time-consuming and attention-diverting nature, are impractical to deploy in certain situations such as those involving physical activity, skilled motor tasks and non-physical tasks involving sustained concentration. Single-item scales also divert attention but the extent to which they do so is much more conducive to the context of exercise. Alternative approaches to measuring fatigue have involved using single item scales that quantify the intensity of the subjective feeling state, usually among clinical populations [33, 35]. Whilst most situation-specific scales are pragmatic and have important applied or clinical applications, a disadvantage is that the data from these instruments are not generalisable and are of only limited use in moving towards a common understanding of fatigue.

An alternative approach to measuring fatigue is to develop a general scale that instead quantifies the intensity of the subjective feeling state, regardless of situational or qualitative variations in feelings of fatigue. That is not to intentionally disregard situation-specific or qualitative differences in fatigue, but rather to adopt a different measurement approach whereby fatigue intensity is decoupled from the type of fatigue in ways conceptually analogous to isolating the brightness from the saturation of a colour. This has the potential to identify and better-understand the interactions between fatigue, behaviour and performance that are common to a variety of situations. The validity, sensitivity and appropriateness of various scales have been comprehensively reviewed elsewhere [36, 37], from which it can be concluded that even very well-cited and popular scales, if used in settings for which they were not designed, can have limitations of the kind previously described. Nevertheless, the fatigue-intensity-rating approach has a number of compelling advantages. Given that most people are likely to experience not one but a range of qualitatively distinct fatigue-like feelings during the course of a day, week, month, year or longer, a general scale of fatigue will make it easier to quantify longitudinal variations in the intensity of their fatigue perceptions and make it simpler to pinpoint events that trigger episodes of fatigue. Furthermore, a general rating scale of fatigue would be a more instant and user-friendly way for individuals to self-monitor fatigue perceptions and regulate behaviours, particularly compared to complex and time-consuming multi-item questionnaires.

A further important distinction to make is between perceptions of exertion and perceptions of fatigue. Gunnar Borg’s earliest, still valid, operational definition of perceived exertion was “…how heavy it feels [to pedal] and … how laborious it feels to work” [38]. In contrast, the subjective perception of fatigue [2, 3] has also been defined as “…the awareness of a decreased capacity for physical and/or mental activity due to an imbalance in the availability, utilization, and/or restoration of resources needed to perform activity” [39]. More recently the nature of perceived exertion has been questioned [16–19], with some pointing out the conceptual and neurological distinction from effort [18] and others providing evidence that physical sensations can be distinguished from a mental sense of effort [17]. Another view is that perceived exertion is the product of central corollary discharge and is independent of afferent feedback [19]. While the current debate regarding the multidimensional nature of perceived exertion and sense of effort is welcome, we also believe that the distinction between perceived exertion, effort and fatigue is equally important. In this regard we put forward several important arguments. First is that perceived exertion, or the subjective experience of how hard a physical task feels, is quite different to perceived fatigue, which we argue is a feeling of diminishing capacity to cope with physical or mental stressors, either imagined or real. Our second assertion is that, while we acknowledge the excellent psychophysical properties of most perceived exertion scales [14, 15, 38, 40, 41], measurements of exertion should only be used for their intended purpose of quantifying how hard a task feels. This suggests that perceived exertion scales should not be used to measure fatigue or fatigability. This point is reinforced by the fact that, while we might expect perceptions of exertion and fatigue to correlate during exercise, once exercise ceases perceived exertion should immediately drop to its lowest point on the scale whereas perceived fatigue should gradually diminish. Consequently, our third argument is that, as a continuous construct experienced at all moments in time, perceived fatigue has better utility than perceived exertion, a discrete construct only experienced during episodes of physical work, in quantifying the readiness, potential or capacity of a person to perform physical or mental work and further work. As such, perceived fatigue measurements have great potential in accounting for intra-individual and situational variations in performance, and in furthering our understanding of the relationship between fatigability, fatigue and the limits of human performance. For example, since fatigue is a continuous feeling state that can be measured at any moment in time, it is possible to imagine that individual variations in fatigue leading up to athletic events might correlate with individual variations in performance or might be a useful indicator of overtraining, poor recovery or any other circumstance likely to impact on performance. Indeed, significant difficulties have been highlighted in the early detection of overtraining syndrome, and it has been noted that no physiological, performance, biochemical or psychological measures have been sufficiently successful in differentiating the condition from other pathologies [42]. The authors specifically call for the development of new diagnostic tools to help detect overtraining syndrome and, in this the regard, ratings of fatigue may be useful.

The points discussed above suggest there is a need for a simple rating scale of fatigue that can be used not only to track sudden changes in subjective perceptions of fatigue intensity during exercise and recovery, but also slower changes in fatigue intensity using the same scalar units across an hourly, daily, weekly or longer time frame. The purpose of the four experiments presented in this manuscript was to develop such a scale, which we have named the ‘Rating-of-Fatigue (ROF) Scale’. In the first experiment we present the conceptual and empirical evidence used to design and construct the ROF scale. In the second experiment we tested the subjective face validity of ROF scale. In the third experiment we objectively measured the convergent and discriminant validity of the ROF scale during both ramped cycling to exhaustion and resting recovery. In the fourth and final experiment we tested the validity of the ROF scale to measure changes in perceived fatigue during longer daily and weekly cycles.

## Experiment 1: Rating-of-Fatigue Scale Development

### Methods

#### Participants

Eighteen healthy adult males (mean age 20.5 ± 0.85 years, height 180 ± 6.5 cm and body mass 43.4 ± 4.9 kg) participated in this study. All participants provided written informed consent and the study was approved by the University of Essex Ethics Committee and carried out in accordance with the Declaration of Helsinki.

#### Design

The purpose of this study was to construct a ROF scale comprising numerical, descriptive and diagrammatic components. This was a correlational study during which participants performed a graded cycling test to volitional exhaustion followed by 30 min of rest. Participants were asked to rate how fatigued they felt on an 11-point numerical scale ranging from zero to ten regularly during graded cycling and recovery. They were also asked to select from a pool of varying written descriptors and diagrams the items that best represented how they felt (see Electronic Supplementary Material Appendix S1). Alignment of the descriptor and diagrammatic components of the ROF scale was determined from the modal selections against the various numerical ratings given. Thus the nature and alignment of the numerical, descriptive and diagrammatic components of the ROF scale were established using empirical data. Participants also provided ratings of perceived exertion (RPE), and physiological measurements of heart rate (HR), blood lactate (BLC), oxygen uptake (*V*O_2_), carbon dioxide production (VCO_2_), respiratory exchange ratio (RER) and ventilation rate (VR) measurements were taken.

#### Procedure

##### Pre-Test Measurements

The age, height and body mass of each participant were recorded. Participants were familiarized with the use of the 6–20 variant of the Rating of Perceived Exertion Scale (RPE) in accordance with the recommendations of Borg [40]. Participants were also familiarised with the 11-point 0–10 numerical scale along with a pool of 12 fatigue descriptors and 12 fatigue diagrams that they would be shown during graded cycling and recovery. Participants were given standardised instructions to select a number, descriptor and diagram that best represented the intensity of their overall feelings of fatigue. In a supine position, resting measurements of HR, BLC, *V*O_2_, VCO_2_, RER and VR were recorded.

##### Cycling Ergometry

Each participant performed a ramped cycling test to volitional exhaustion on an electromagnetically braked Lode Excalibur Sport Cycle Ergometer, (Lode, Groningen, The Netherlands). The geometry of the Lode was set for each individual participant so that, with one of the pedals positioned bottom dead centre with the sole of foot parallel to the ground, the knee was flexed at approximately 170°–175°. A ramped protocol was used in which the initial intensity of 10 W was increased by 1 W every 4 s. Participants were asked to pedal at 70 revolutions per minute and the test was terminated at volitional exhaustion, defined in this study as the moment participants could no longer maintain that cadence.

##### Physiological Measurements

Physiological measurements were taken at rest, during the ramped cycling protocol and during a 30-min recovery period during which participants remained seated on the ergometer. Continuous measurements of HR were made using a Polar s610i heart-rate monitor and wireless chest strap (Polar, Finland). Participants were fitted with an appropriate-sized face mask (Hans Rudolph, Kansas City, KS, USA) and breath-by-breath ventilation and gas exchange measurements (*V*O_2_, VCO_2_, RER and VR) were made using an Oxycon CPX (Jaeger, Würzburg, Germany) calibrated in accordance with the manufacturer’s instructions. Capillary blood samples (20 µl) were taken from the earlobe at rest, every 100 s during the cycling test, and then every 2 min during the first 10 min of recovery and every 5 min for the last 20 min of recovery. Lactate concentration was measured enzymatic-amperometrically (Ebio+, Eppendorf, Germany).

##### Psychophysical Measurements

Participants were asked to indicate the RPE, numerical rating of fatigue, fatigue descriptor and fatigue diagram that best represented how they felt at various moments during exercise and recovery. Ratings and selections were taken every 100 s during the cycling test, every 2 min for the first 10 min of recovery, and then every 5 min for the last 20 min of recovery. The RPE scale, numerical fatigue scale, fatigue descriptors and fatigue diagrams were always presented on a separate sheet and in a counterbalanced order. There were 12 different descriptors and 12 different diagrams that reflected varying levels of fatigue. The 12 descriptor options where developed by the authors by incorporating common adjectives found in the previously cited fatigue literature, which were ‘fatigued’, ‘exhausted’, ‘invigorated’ and ‘fresh’. Diagrammatic options were developed to ensure that different fatigue states were adequately represented and that variation in the diagrammatic representation was available. The 12 descriptor and diagrammatic options presented to participants are given in Electronic Supplementary Material Appendix S1. On each presentation sheet the descriptors and diagrams were scattered so as not to be in escalating order. The intention was to create sufficient item choice for participants but not so much choice that each rating took too much time. This would also provide a basis upon which to reduce the number of descriptors and diagrams included in the scale according to modal items selected at particular intensities. On each presentation, participants were prompted if necessary to ensure all four ratings were completed within 30 s, i.e. allowing just under 8 s per scale.

#### Statistical Analysis

##### Rating-of-Fatigue Scale Composition

Respiratory gas exchange measurements and HR were averaged for the last 20 s of every 100-s segment during the cycling test, the last 20 s of every 2 m for the first 10 min of recovery, and then the last 20 s of every 5 min for the last 20 min of recovery. In order to determine the most common alignment of descriptor and diagrammatic components against the 11-point numerical scale, all participants’ exercising and resting responses were pooled together for the numerical item clusters of 0, 2–3, 4–6, 7–8 and 10. This was done to ensure that the descriptors and diagrams represented numerical rating bands for the lower middle and upper range of the scale, and individual numerical ratings for the extreme lower and upper ends of the scale. The modal item was identified and selected for each cluster.

##### Component Correlations for the Rating-of-Fatigue Scale During Exercise

For each individual participant a Pearson’s Product Moment Correlation was calculated for each ROF component against power output, RPE, HR, BLC, *V*O_2_, VCO_2_, RER, VR, time (*t*) and time to exhaustion (TTE), which was calculated as momentary time subtracted from completion time. The resulting individual *r* values were subjected to a single-sample *t* test across the participant group, which revealed whether the correlations were significantly greater or less than zero. An alpha level of 0.05 was used to indicate statistical significance.

### Results

#### Modal Fatigue Descriptors and Diagrams

There were a total of 68 descriptor and diagrammatic selections for the zero ROF increment, 95 selections of the 2–3 ROF band, 94 for the 4–6 ROF band, 41 for the 7–8 ROF band and 14 for the ROF increment of 10. Modal descriptors accounted for 44, 45, 50, 39 and 29% of all selections for the ROF bands of 0, 2–3, 4–6, 7–8 and 10, respectively. For ROF band 10 there were three modal descriptors, each constituting 29% leaving a choice between: (i) 99% fatigued, (ii) extremely fatigued and (iii) total fatigue and exhaustion—nothing left. ‘Extremely fatigued’ was discarded because it was considered too similar to the modal response for ROF band 7–8. ‘Total fatigue and exhaustion—nothing left’ was selected over ‘99% fatigue’ because it was felt the inclusion of two adjectives was a more absolute statement, less likely to be misinterpreted. Modal diagrams accounted for 44, 24, 32, 34 and 57% of all selections for the ROF bands of 0, 2–3, 4–6, 7–8 and 10, respectively. Selection frequency for all 12 descriptors and diagrams are presented in Table 1 with the modal item highlighted for each of the five ROF bands. The ROF scale, as derived from the modal data reported, is presented in “Appendix A”, along the standard ROF instructions that provide participants with information about how to use the scale.

**Table 1:**
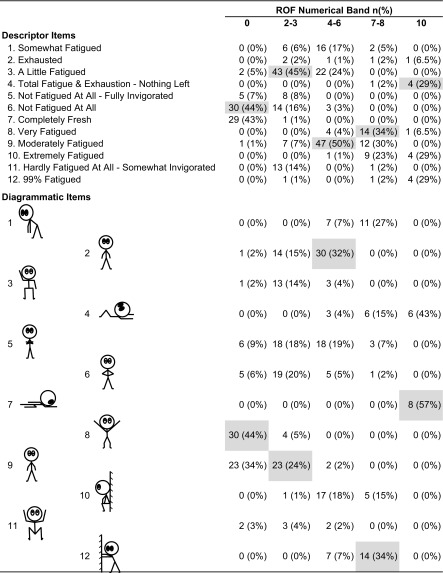
Descriptor and diagramatic selection frequency, *n* (%), that correspond with the numeric bands of the rating-of-fatigue (ROF) scale. Modal items selected for inclusion in the final version of the ROF scale are highlighted

#### Rating-of-Fatigue Component Correlations with Performance, Physiological and Psychophysical Constructs

The means of individual participant correlation coefficients calculated between numerical ROF and various performance, physiological and psychophysical constructs measured during the graded cycling task to exhaustion were all greater than 0.900 with the exception of RER which was 0.894. Mean correlation coefficients during recovery were also all very high and ranged between 0.767 and 0.888. Single-sample *t* test outcomes showed a negative correlation for time to exhaustion, and positive correlations for all other constructs that were significantly different to no correlation. Very large effect sizes of  >0.9 were observed for all measures. Detailed correlation and single-sample *t* test outcomes are presented in Table 2.

**Table 2:** Mean Pearson product moment correlation coefficients between numeric rating of fatigue and various performance, physiological and psychophysiological constructs (Experiment 2)

	Mean Pearson coefficients		Single sample *t* test outcomes		
	*r*_MEAN_	SD of *r*_MEAN_	*t*(17)	*P*	*η* ^2^
Graded exercise					
Rating of perceived exertion	0.991	0.007	600	<0.0001	0.999
Heart rate	0.973	0.053	78	<0.0001	0.997
*V*O_2_	0.979	0.022	188	<0.0001	0.999
VCO_2_	0.981	0.018	228	<0.0001	0.999
Blood lactate concentration	0.969	0.033	124	<0.0001	0.998
Respiratory exchange ratio	0.894	0.143	27	<0.0001	0.976
Ventilation rate	0.979	0.022	191	<0.0001	0.999
Power output	0.992	0.005	920	<0.0001	0.999
Time to exhaustion	−0.992	0.005	−920	<0.0001	0.999
Recovery					
Heart rate	0.856	0.206	18	<0.0001	0.948
*V*O_2_	0.795	0.121	28	<0.0001	0.978
VCO_2_	0.878	0.077	48	<0.0001	0.992
Blood lactate concentration	0.818	0.216	16	<0.0001	0.937
Respiratory exchange ratio	0.888	0.094	40	<0.0001	0.989
Ventilation rate	0.767	0.128	25	<0.0001	0.974

Ratings of perceived exertion, power output and time to exhaustion are omitted from the recovery section of the table since they are only relevant to exercise

*r*_MEAN_ constitutes the mean of all correlation coefficients calculated for each individual participant, *SD* 1 standard deviation; single-sample *t* test outcomes are presented to show the extent to which coefficients are greater or less than zero, *η* ^2^ eta-squared effect size

## Experiment 2: Face Validity of the Rating-of-Fatigue Scale

### Methods

#### Participants

Male (*n* = 59) and female (*n* = 44) participants from the University of Essex were recruited for this study. These included sport and exercise science academics (*n* = 10), academics from non-sport disciplines (*n* = 10), undergraduate sports science students (*n* = 36) and postgraduate students (*n* = 47). All of the participants were selected because of their varying levels of expertise in sport and exercise science, and because of their familiarity with participating in physical activity, exercise and sport. All participants provided their written informed consent to take part in the study, which was subject to institutional ethical approval and carried out in accordance with the Declaration of Helsinki.

#### Design

The purpose of this study was to test the face validity of the ROF scale (Supplement 1) derived from the data presented in Experiment 1. Face validity is a subjective test of whether an instrument appears to measure what it purports to, which for the ROF scale is the perceived level of fatigue. In this study, participants were asked to rate what they thought the ROF scale measured using both open and closed questionnaire methods. Responses were recorded before and after participants read the scale instructions (Supplement 1) to determine whether the instructions improved participants’ comprehension and intended purpose of the ROF scale.

#### Procedure

Participants were first presented with the ROF scale to inspect without instructions. They were then asked to respond to eight questionnaire items by rating them on a five-point Likert scale (Strongly agree; Agree; Undecided; Disagree; Strongly Disagree) according to the extent to which the ROF: (i) represents fatigue, (ii) represents exertion, (iii) descriptive components make the scale easy to understand, (iv) descriptive components assist in deciding upon a rating, (v) diagrammatic components make the scale confusing, (vi) diagrammatic components assist in deciding upon a rating, (vii) overall scale is difficult to understand, and (viii) visual appearance is appealing. Once participants had provided their responses, they were asked to re-inspect the ROF scale again, this time after reading the accompanying instructions (Supplement 1). Participants were then asked to respond to the eight previously described Likert items plus an additional item about the usefulness of the instructions in understanding the scale. These procedures are consistent with guidelines on face validity testing [43].

#### Statistical Analysis

All questionnaire Likert scale responses were scored from 0 to 4, such that 0 represented low face validity and 4 represented high face validity. Face validity questionnaire item scores, before and after administration of the ROF scale instructions, were compared using non-parametric Wilcoxon’s signed rank tests. All outcomes are presented as mean  ±  1 SD and an alpha level of <0.05 to indicate statistical significance. Eta-squared effect sizes (*η* ^2^) are given.

### Results

A high level of face validity for the ROF scale was found, as indicated by a high mean Likert score, implying that the scale could measure fatigue. This score increased further after the scale instructions had been read (pre 3.5 ± 0.6 vs. post 3.7 ± 0.6, *Z*_104_ = −2.2, *P* = 0.013, *η* ^2^ = 0.05). Likert scores indicated participants were initially undecided about the extent to which the scale represented exertion (2.0 ± 1.2), but this improved slightly after reading the instructions (1.7 ± 1.3) (*Z*_104_ = −2.8, *P* = 0.002, *η* ^2^ = 0.08).

High Likert scores, which did not change significantly after reading the instructions, indicated that the ROF scale descriptors were perceived to help clarify the scale (pre 3.5 ± 0.6 vs. post 3.5 ± 0.6, *Z*_104_ = −0.2, *P* > 0.05, *η* ^2^ < 0.01) and help in making ratings (pre 3.4 ± 0.7 vs. post 3.4 ± 0.7, *Z*_104_ = −0.7, *P* > 0.05, *η* ^2^ < 0.01). Similarly, Likert scores related to the ROF diagrams did not change after reading the instructions but, although the weakest of the face validity outcomes, were still perceived to help make ratings (pre 2.7 ± 1.1 vs. post 2.7 ± 1.1, *Z*_104_ = −0.3, *P* > 0.05, *η* ^2^ < 0.01) without causing confusion (pre 1.2 ± 1.0 vs. post 1.1 ± 1.1, *Z*_104_ = −1.7, *P* > 0.05, *η* ^2^ = 0.03).

**Fig. 1:**
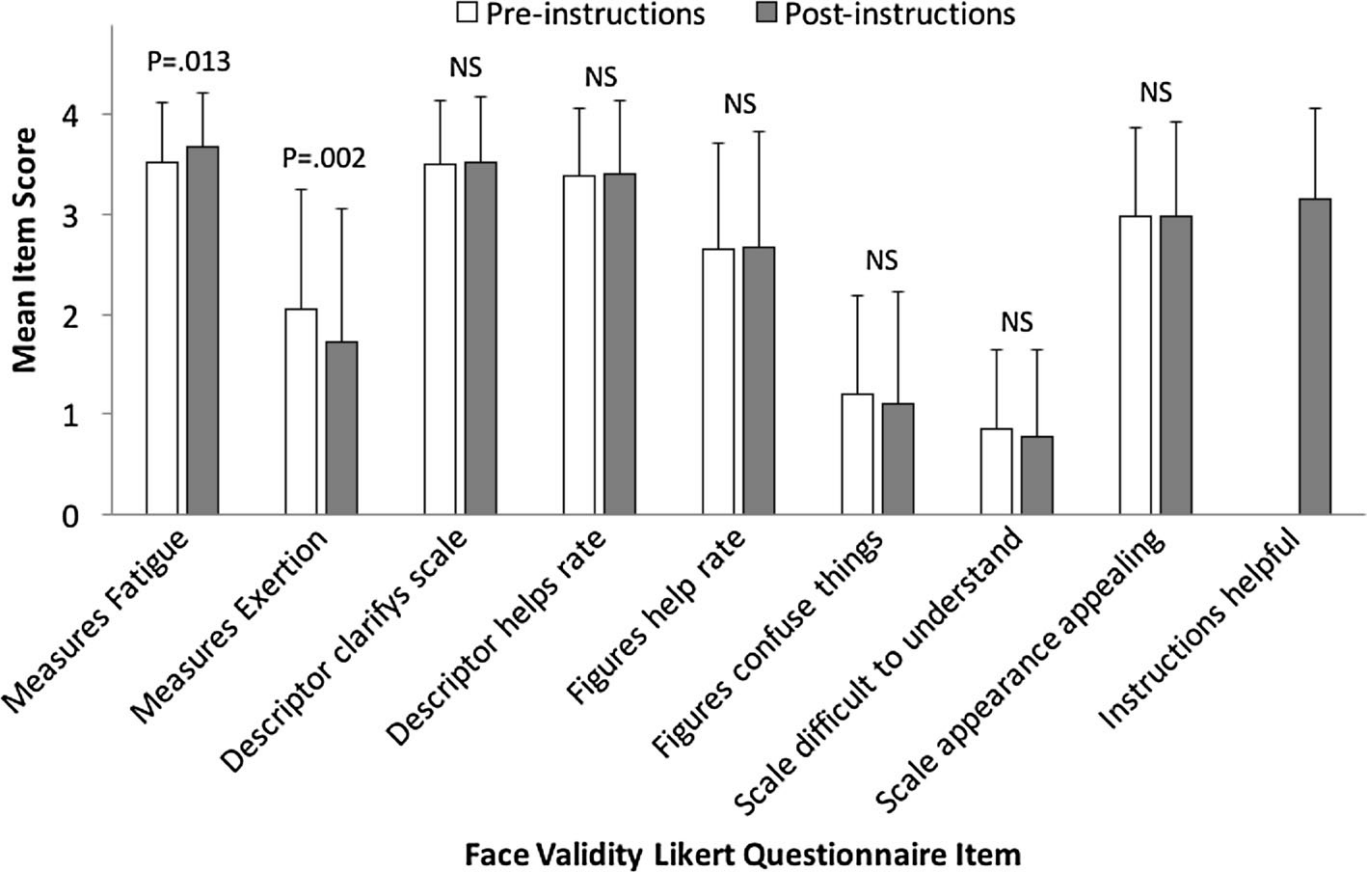
Face validity outcomes of the rating-of-fatigue scale before and after reading the scale instructions

Both before and after reading the instructions, participants agreed that the ROF scale was visually appealing (pre 3.0 ± 0.9 vs. post 3.0 ± 0.9, *Z*_104_ = −0.05, *P* > 0.05, *η* ^2^ < 0.01) and disagreed that it was difficult to understand (pre 0.9 ± 0.8 vs. post 0.8 ± 0.8, *Z*_104_ = −0.9, *P* > 0.05, *η* ^2^ < 0.01). The ROF scale instructions were rated as being useful (3.1 ± 0.8). Comparisons of pre- and post-instruction face validity Likert scale scores for the ROF scale are presented in Fig. 1.

## Experiment 3: Convergent and Discriminant Validity of the Rating-of-Fatigue Scale During Ramped Cycling to Volitional Exercise and Resting Recovery

### Methods

#### Participants

Twenty healthy male participants (mean age 20.5 ± 0.82 years, height 180 ± 0.6 cm and body mass 43.6 ± 4.7 kg) were recruited for this study. The study was subject to institutional ethical approval and carried out in accordance with the Declaration of Helsinki. Written informed consent was obtained from all participants.

#### Design

The purpose of this study was to carry out an objective evaluation of the construct validity of the ROF scale. Much of the design was the same as Experiment 1 except that the finalized and complete version of the ROF scale was presented to participants rather than isolated numerical, descriptor and diagrammatic components. Owing to the issues described with other scales of fatigue, particularly regarding their use during exercise, such scales were not incorporated into the present study. Instead, attempts were made to establish convergent validity during cycling with other objective physiological and performance measures. Two objective testing methods were used during both ramped exhaustive cycling and 30 min of resting recovery: (i) convergent validity in which associations were made between ROF measurements and various physiological and performance markers of fatigue; (ii) discriminant validity by measuring the degree to which ROF and RPE diverge. All measurements were taken during a single laboratory attendance.

#### Procedure

##### Pre-Test Measurements

Prior to testing all participants were familiarised with the use of both ROF and RPE scales. In a resting supine position HR, BLac, *V*O_2_, VCO_2_, RER and VR were recorded in the same way as described for Experiment 1.

##### Cycling Ergometry, Resting Recovery and Physiological Measurements

All participants performed a ramped cycling test to volitional exhaustion followed by 30 min of resting recovery. During exercise and rest continuous measurements of HR, BLC, *V*O_2_, VCO_2_, RER and VR were recorded. The cycling ergometry, resting recovery and physiological testing methods were exactly the same as described for Experiment 1.

##### Psychophysical Measurements

During the ramped cycling test participants were asked to provide a ROF and RPE every 100 s which equated to each 25-W increase in workload. During the resting recovery ROF and RPE measurements were taken every 2 min for the first 10 min of recovery, then every 5 min for the last 20 min of recovery.

#### Statistical Analysis

Respiratory gas exchange measurements and HR were averaged for the last 20 s of every 100-s segment during the cycling test, the last 20 s of every 2 min for the first 10 min of recovery, and then the last 20 s of every 5 min for the last 20 min of recovery. All variables are expressed in relation to the percentage of time to exhaustion, whereby 0% represents the beginning of the ramped cycling test and 100% represents the point of volitional exhaustion which, in absolute terms, differed between participants. In order to provide a continuous scale of time during cycling and recovery, recovery time was also expressed as a percentage of time to exhaustion whereby the point of fatigue occurred at 100% and recovery time as a percentage increase in time relative to time to exhaustion. For example, if a participant terminated the exercise test at 30 min, 30 min of recovery would end at 200% time to exhaustion.

For each participant, a Pearson’s Product Moment Correlation was calculated for each ROF measure against the RPE, and each of the measured variables (HR, BLC, *V*O_2_, VCO_2_, RER and VR). The resulting individual *r* values were subjected to a single-sample *t* test across the participant group, which revealed whether the correlations were significantly greater or less than zero. An alpha level of 0.05 was used to indicate statistical significance and effect sizes are presented as eta-squared (*η* ^2^).

### Results

During the cycling test strong correlations were found between the ROF scale and the following measurements: (i) RPE, (ii) HR, (iii) *V*O_2_, (iv) VCO_2_, (v) BLC, (vi) RER, (vii) VR, (viii) power output and (ix) time to exhaustion. This indicates the ROF scale has high levels of convergent validity during exercise with various performance, psychophysical and physiological constructs.

High levels of convergent validity during recovery were also found, as indicated by significant correlations between the ROF scale and HR, *V*O_2_, VCO_2_, BLC, RER and VR. During recovery the ROF scale exhibited divergent validity against RPE, and correlation calculations were not possible since all recorded RPE scores were 6 without any variance.

**Fig. 2:**
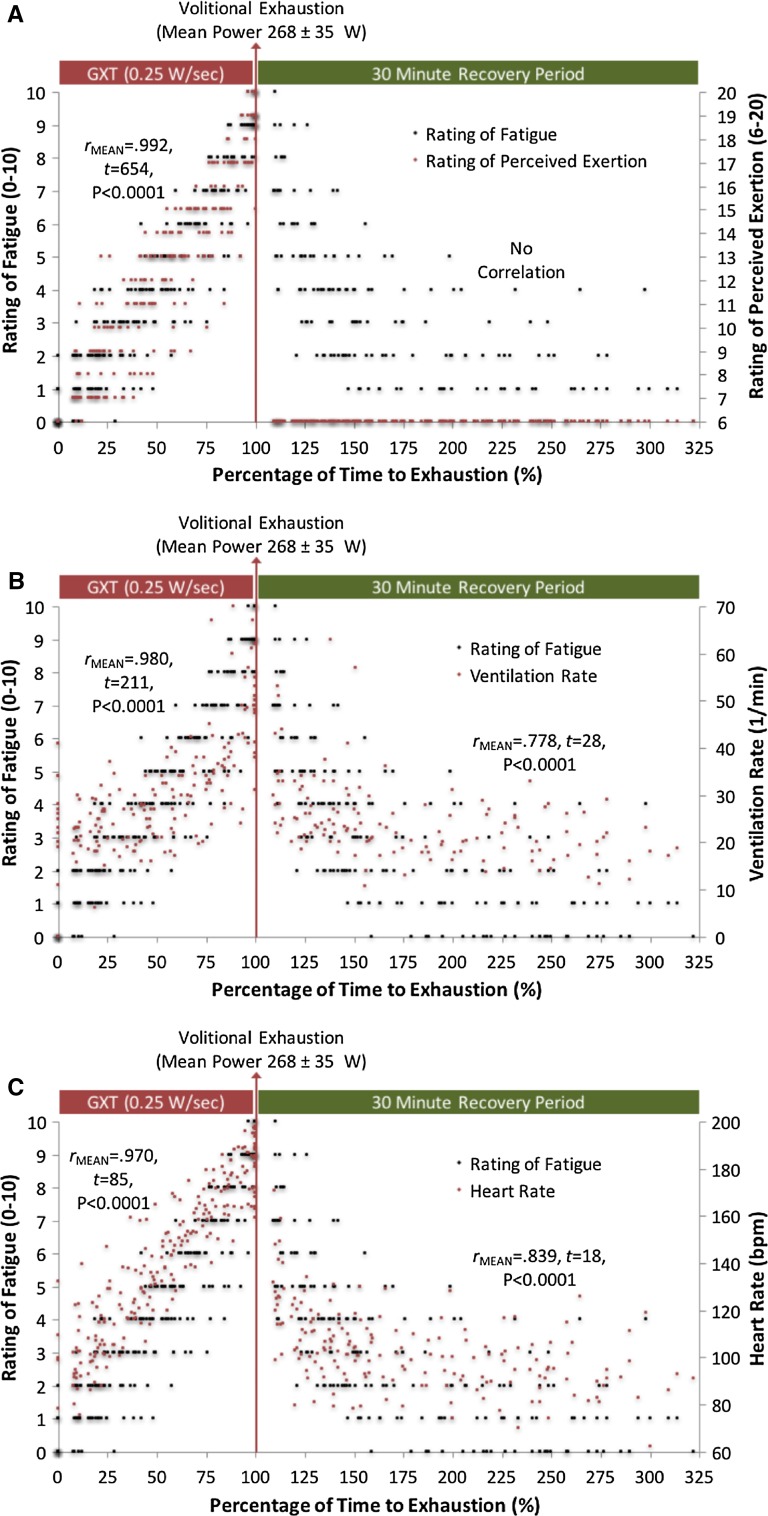
Relationship between ratings of fatigue and perceived exertion (**a**), ventilation rate (**b**) and heart rate (**c**) during graded cycling to exhaustion and 30 min of resting recovery

**Fig. 3:**
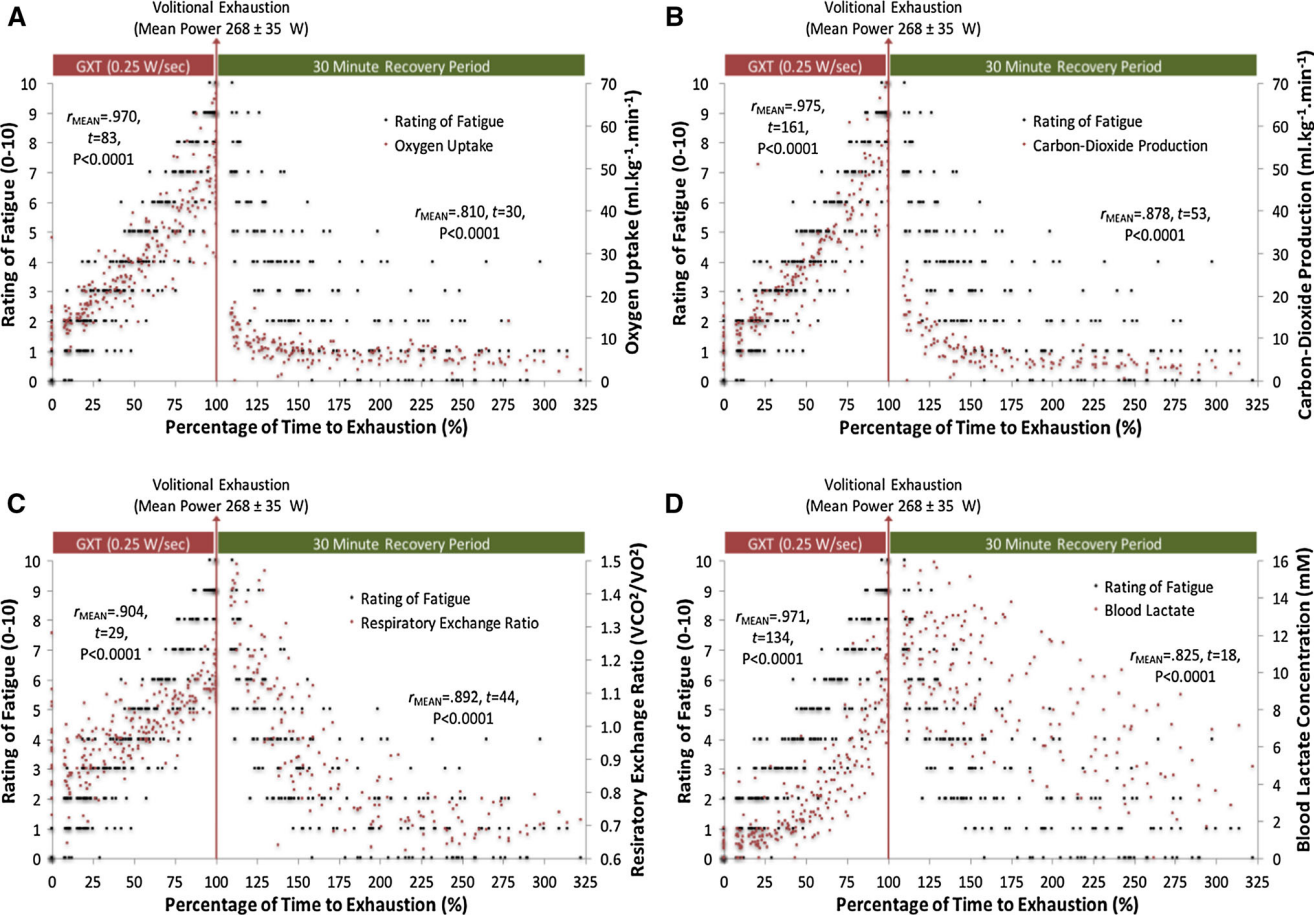
Relationship between ratings of fatigue and oxygen uptake (**a**), carbon dioxide production (**b**), respiratory exchange ratio (**c**) and blood lactate concentration (**d**) during graded cycling to exhaustion and 30 min of resting recovery

Mean and SD Pearson Product Moment correlation coefficients together with single-sample *t* test outcomes and effects sizes are given in Table 3. ROF and RPE associations during graded cycling and recovery are presented in Fig. 2a. ROF convergent validity with VR and HR is presented in Fig. 2b, c, respectively. ROF convergent validity with VO_2_, VCO_2_, RER and BLC are presented in Fig. 3a–d, respectively.

**Table 3:** Mean Pearson product moment correlation coefficients between numeric rating of fatigue and various performance, physiological and psychophysiological constructs (Experiment 3)

	Mean Pearson coefficients		Single sample *t* test outcomes		
	*r*_MEAN_	SD of *r*_MEAN_	*t*(19)	*P*	*η* ^2^
Graded exercise					
Rating of perceived exertion	0.992	0.007	654	<0.0001	0.999
Heart rate	0.970	0.051	85	<0.0001	0.997
*V*O_2_	0.970	0.052	83	<0.0001	0.997
VCO_2_	0.975	0.027	161	<0.0001	0.999
Blood lactate concentration	0.971	0.032	134	<0.0001	0.999
Respiratory exchange ratio	0.904	0.139	29	<0.0001	0.978
Ventilation rate	0.980	0.021	211	<0.0001	0.999
Power output	0.966	0.028	152	<0.0001	0.999
Time to exhaustion	−0.921	0.204	−20	<0.0001	0.995
Recovery					
Heart rate	0.839	0.204	18	<0.0001	0.945
*V*O_2_	0.810	0.124	30	<0.0001	0.979
VCO_2_	0.878	0.075	53	<0.0001	0.993
Blood lactate concentration	0.825	0.206	18	<0.0001	0.945
Respiratory exchange ratio	0.892	0.090	44	<0.0001	0.990
Ventilation rate	0.778	0.127	28	<0.0001	0.976

Ratings of perceived exertion, power output and time to exhaustion are omitted from the recovery section of the table since they are only relevant to exercise

*r*_MEAN_ constitutes the mean of all correlation coefficients calculated for each individual participant, *SD* 1 standard deviation; single-sample *t* test outcomes are presented to show the extent to which coefficients are greater or less than zero, *η* ^2^ eta-squared effect size

## Experiment 4: Convergent Validity of Circadian and Circaseptan Variations in Ratings of Fatigue

### Methods

#### Participants

Fifty participants were recruited for this study comprising 37 males and 13 females (mean age 32.3 ± 9.7 years, height 175.3 ± 9.9 cm, body mass 74.6 ± 14.4 kg and body mass index 24.1 ± 3.5 kg/m^2^). Only healthy participants were recruited, those suffering from any injury, disease, illness or mental health condition were excluded from the study. Those taking prescribed medication were also excluded from the study, as were shift-workers and weekend workers.

Participants provided written informed consent to undertake the procedures used in this study which approved by the institutional ethics committee and carried out in accordance with the Declaration of Helsinki.

#### Design

The purpose of this study was to validate ROF during daily and weekly living and working activity cycles. A 7-day longitudinal study design was used during which, during waking hours, participants continually wore a tri-axial accelerometer to objectively measure activity and provided ratings of fatigue corresponding with key daily activities.

#### Procedure

##### Pre-Test Procedures

Participants attended the laboratory before the 7-day data capture period and were familiarised with the ROF scale and the use of the accelerometer. Participants were asked not to make any major changes to their living or working routines during the 7-day capture period, for example not to suddenly change their working hours or to suddenly take up or increase physical activity levels. Each participant took away with them a copy of the ROF scale (Supplement 1) and an accelerometer that was initialised and set to record for 7 consecutive days.

##### Ratings of Fatigue and Daily Recording Segments

Each participant was familiarised with the ROF and asked to provide ratings at standard points each day for 7 days. Each day was broken up into segments that corresponded with key daily events that most people commonly experience. The daily segments differed slightly for working days (Monday–Friday) and non-working days (Saturday and Sunday) to take into account travelling to and from work. The ROF recording moments were: (i) upon waking, (ii) 10 min after waking, (iii) arriving at work (not weekend variant), (iv) before lunch, (v) after lunch, (vi) before leaving work (not weekend variant), (vii) arriving home (not weekend variant), (viii) before dinner (not weekday variant) and (ix) bedtime.

##### Accelerometer Measurements

Each participant wore an ActiGraph GT1M (ActiGraph, LLC, Fort Walton Beach, FL, USA) tri-axial accelerometer during this study. Participants were instructed to wear the accelerometer, in accordance with the manufacturer’s instructions, positioned on a lateral aspect of the waist for a continuous period of 7 days except when sleeping, showering or bathing. A relatively short recording epoch of 15 s was selected to potentiate detailed interrogation of particular short-term events occurring within the 7-day capture period. Raw activity counts recorded by the accelerometer were used in this study because they represent continuous data suitable for correlating with ROF, rather than the discrete category systems of physical activity level often associated with accelerometer research. Non-wearing time was defined as bouts of ≥60 min of consecutive zero counts and these periods were removed from the analysis [44].

#### Statistical Analysis

Pearson’s Product Moment Correlation tests were used to examine the relationship between ROF, time of day and accumulated segment accelerometer count data for each participant for each day. The resulting individual *r* values were subjected to a single-sample *t* test across the participant group, which revealed whether the correlations were significantly greater or less than zero. Paired samples *t* tests were used to determine the differences between averaged ROF for each day and the weekly average ROF. In all tests, an alpha level of 0.05 was used to indicate statistical significance and effect sizes are reported as eta-squared (*η* ^2^).

### Results

**Fig. 4:**
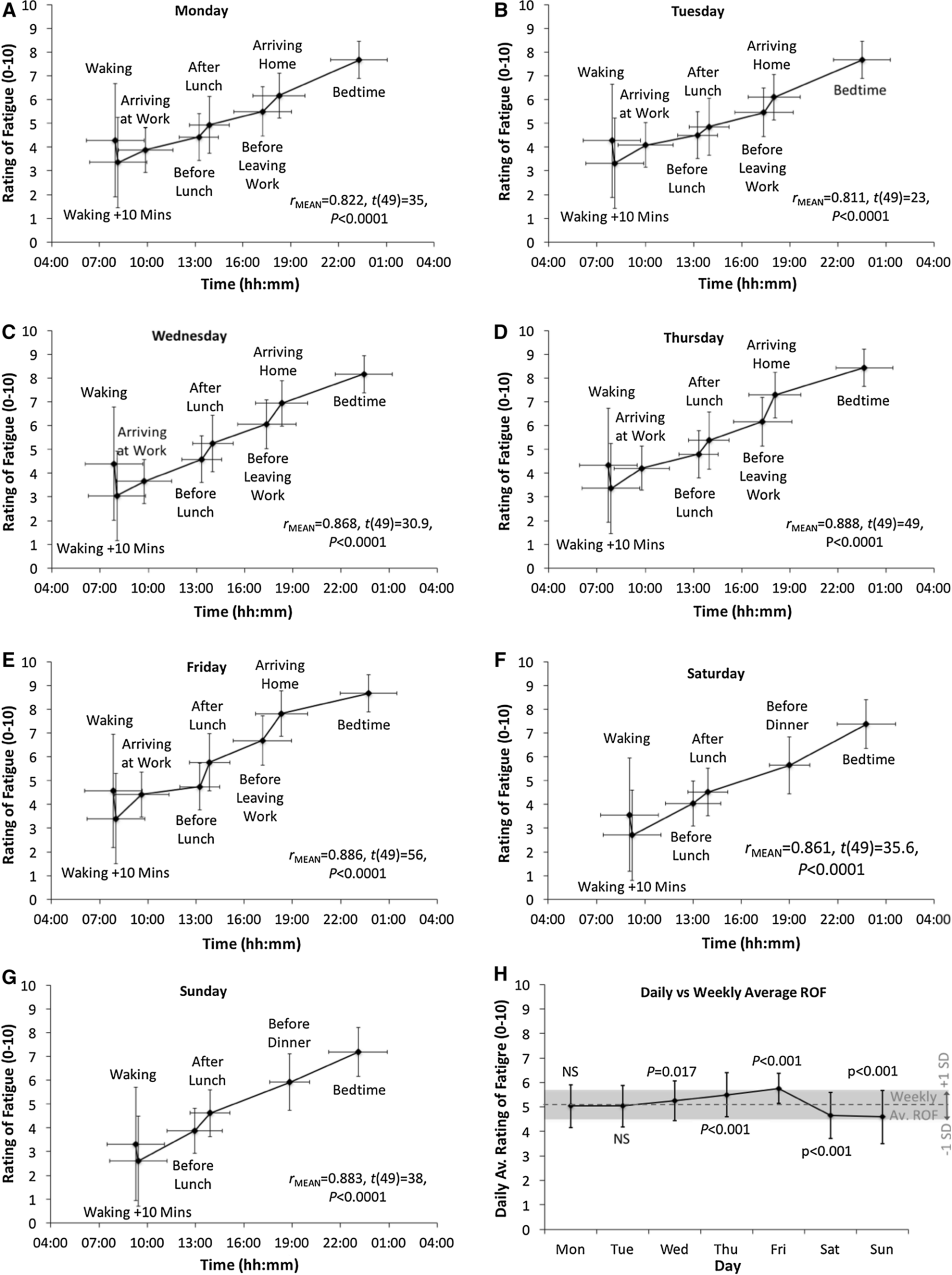
Relationship between ratings of fatigue and daily changes in time from Monday to Sunday (**a**–**g**). Comparison of daily and weekly average ratings of fatigue (**h**)

Strong associations between ROF and time of day were found for all days of the working week (Monday to Friday) and the weekend (Fig. 4a–g). This indicates strong convergent validity of the ROF with daily time-related fatigue.

Paired-samples *t* tests found daily average ROF did not differ compared to weekly average for Monday (5.0 ± 0.9 vs. 5.1 ± 0.6, *t*_49_ = −0.8, *P* = 0.44, *η* ^2^ = 0.013) or Tuesday (5.0 ± 0.8 vs. 5.1 ± 0.6, *t*_49_ = −1.3, *P* = 0.20, *η* ^2^ = 0.033). However, average daily ROF was higher than the weekly average for Wednesday (5.3 ± 0.8 vs. 5.1 ± 0.6, *t*_49_ = −2.2, *P* = 0.017, *η* ^2^ = 0.090), Thursday (5.4 ± 0.9 vs. 5.1 ± 0.6, *t*_49_ = −4.5, *P* < 0.001, *η* ^2^ = 0.292) and Friday (5.8 ± 0.6 vs. 5.1 ± 0.6, *t*_49_ = 8.6, *P* < 0.001, *η* ^2^ = 0.601). Average daily ROF was lower than the weekly average for Saturday (4.6 ± 0.9 vs. 5.1 ± 0.6, *t*_49_ = −5.2 *P* < 0.001, *η* ^2^ = 0.356) and Sunday (4.5 ± 1.06 vs. 5.1 ± 0.6, *t*_49_ = −5.6, *P* < 0.001, *η* ^2^ = 0.390). Differences between daily and weekly average ratings of fatigue are presented in Fig. 4h.

**Fig. 5:**
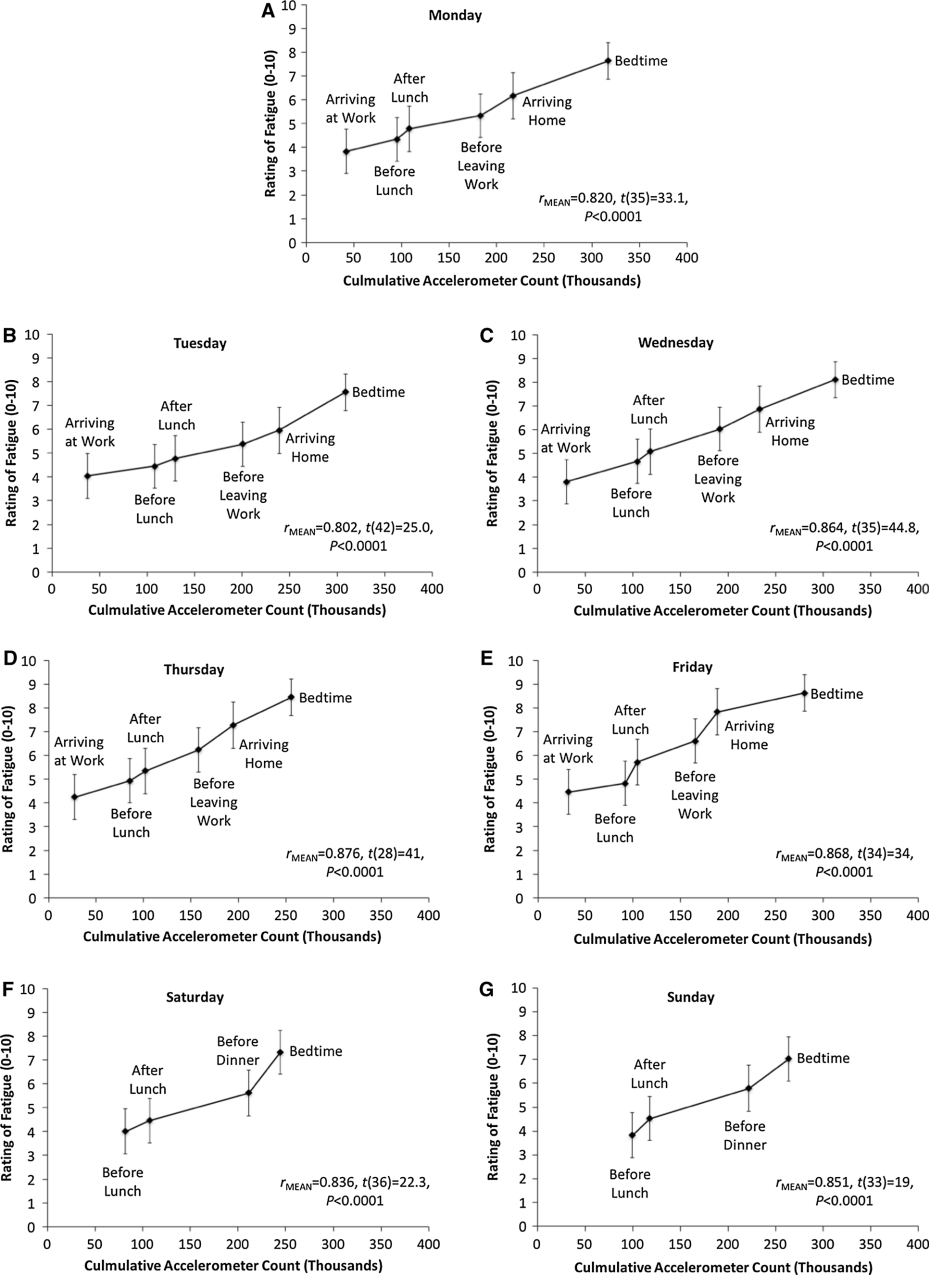
Relationship between ratings of fatigue and daily accumulated accelerometer count from Monday to Sunday (**a**–**g**)

Considerable variation was observed in the time participants put on their accelerometers after waking, which seems due to different cleaning and dressing schedules. To compensate for this and the associated unreliability of early morning accelerometer count, the first correlation point for weekdays was arriving at work and for weekends was before lunch. Strong associations were found between ROF and segment accumulated accelerometer count every day of the week (Fig. 5a–g.) All coefficients, single-sample *t* test outcomes and effect sizes for the associations between ROF, time of day and cumulative accelerometer count are given in Table 4.

**Table 4:** Mean Pearson product moment correlation coefficients between numeric rating of fatigue and time of day, and cumulative accelerometer count

	Mean Pearson coefficients		Single sample *t* test outcomes			
	*r*_MEAN_	SD of *r*_MEAN_	*df*	*t*	*P*	*η* ^2^
ROF—time of day association						
Monday	0.822	0.165	49	35	<0.0001	0.980
Tuesday	0.811	0.245	49	23	<0.0001	0.915
Wednesday	0.868	0.199	49	31	<0.0001	0.951
Thursday	0.888	0.126	49	50	<0.0001	0.981
Friday	0.886	0.112	49	56	<0.0001	0.985
Saturday	0.861	0.170	49	36	<0.0001	0.964
Sunday	0.883	0.164	49	38	<0.0001	0.967
ROF—accelerometer count association						
Monday	0.820	0.148	35	33	<0.0001	0.957
Tuesday	0.802	0.210	42	25	<0.0001	0.927
Wednesday	0.684	0.116	35	45	<0.0002	0.976
Thursday	0.876	0.115	28	41	<0.0003	0.972
Friday	0.868	0.149	34	34	<0.0001	0.959
Saturday	0.836	0.227	36	22	<0.0001	0.908
Sunday	0.851	0.254	33	19	<0.0001	0.880

*r*_MEAN_ constitutes the mean of all correlation coefficients calculated for each individual participant, *SD* 1 standard deviation; single-sample *t* test outcomes are presented to show the extent to which coefficients are greater or less than zero, *η* ^2^ eta-squared effect size

## Discussion

The series of studies we have reported show that the new ROF scale we have developed has good face validity and high levels of convergent validity with various constructs of fatigue during ramped cycling to exhaustion, resting recovery and daily living activities. We also observed ROFs diverge from perceived exertion during resting recovery, establishing the discriminant validity of the ROF scale. Unlike the various well-established perceived exertion scales [14, 15, 34, 39, 40] that are designed for isolated use during physical activity, the ROF scale is an instrument capable of tracking perceptions of fatigue across any range of living, physical activity and recovery contexts. As such the scale has applied and heuristic potential in perceptual monitoring as an effective component of strategies designed to prevent overtraining, over-reaching, injury and illness [25, 45–47] as well as evaluating athletes’ readiness to perform or repeat physical tasks. The ability to track fatigue using a continuous measure through training and post-exercise recovery could be an advantageous, simple and sensitive instrument for detecting overtraining syndrome. The scale also exhibited a high degree of validity in tracking changes in fatigue that may progressively increase throughout the course of a day and the course of a week. The ROF scale may have utility for sufferers of certain chronic illnesses who need to quickly monitor and regulate their activities as a way of managing fatigue symptoms.

Experiment 2 showed that, both with and without the instructions, the ROF scale had high face validity. The accompanying descriptors and diagrams were found to help participants understand the scale even in the absence of the instructions. The instructions were found to improve participants’ ability to use the scale and make ratings. The instructions also helped participants distinguish the constructs of fatigue from exertion, although the higher than expected scores returned for this item indicated some uncertainty about the extent to which the scale measured exertion. As such, we advocate that prior to using the ROF scale, all participants should be familiarised with its use which should involve visually inspecting the scale and reading the corresponding instructions (“Appendix A”). Participants should also be given the opportunity to ask questions and clarify any uncertainties they may have.

As expected, ROF during exercise was found to strongly correlate with RPE and various physiological markers during Experiment 3. Perhaps what is most interesting is the correlation found between ROF and physiological markers during recovery in circumstances where RPE remained at 6 (lowest). While RPE was never intended to track perceptions during recovery, there are advantages of doing so which the ROF has been found to do well. The ROF may be a useful field method to track the speed at which an athlete is recovering, in situations where it is impractical, expensive or ineffective to monitor physiological changes. It must be noted that in this study we have presented linear correlational outcomes during recovery but acknowledge that, similar to the pattern of change commonly seen in many physiological parameters during recovery, the reduction in perceived fatigue may also be non-linear and this relationship warrants further investigation.

The ability to continuously measure ratings of fatigue before, during and after exercise using a single scale could be efficacious in understanding intra-individual variations in the performance of a task carried out at different times in seemingly identical circumstances. For example, during training athletes sometimes experience variations in performance that cannot be explained in terms of differences to their nutrition, time of training, recovery interval, weather or other factors. This has also been a long recognised challenge in experimental studies and the reason why researchers go to extraordinary lengths to standardise conditions in repeated-measures studies, for example, standardizing the diet, training, sleep, environmental conditions and lab attendance time. The ROF scale could therefore have a confirmative purpose in measuring the effectiveness of such standardisation methods to ensure participants have comparative levels of readiness to perform between attendances or tasks. If a large difference in pre-test ROF is detected, potentially participants could be asked to return on another day or be withdrawn from the study. Alternatively, pre-test ROF could be used through covariation analysis to partial out individual fluctuations in perceived fatigue when investigating performance effects.

In Experiment 4, the key findings were that ROF gradually increased each day from waking to going to bed. These daily increases correlated very strongly with accumulated accelerometer count, supporting the expected relationship between daily activity levels and perceptions of fatigue. Furthermore, daily average ROF, when compared to the weekly average ROF, was higher from Wednesday onwards and progressively increased on Thursday and Friday. Saturday and Sunday daily averages were however significantly lower. While these results confirm what might intuitively be expected, they nevertheless highlight the fatiguing effect the working week has on individuals and the restorative importance of regular non-working intervals such as the weekend. The longer term negative effects of long working conditions of various health outcomes are known [48], and the ROF may be useful in investigating how personal resources moderate the relationship between work demand and fatigue and well-being outcomes [49]. It is also acknowledged that gradual increases in fatigue seen throughout the day could in some circumstances be counteracted by exercise or other invigorating activities. The ROF has great potential in developing a better understanding of the role exercise can play in reducing the symptoms of fatigue.

### Intensity-Based Approaches to Measuring and Monitoring Perceived Fatigue

The ROF reveals nothing about qualitative distinct variations in perceptions of fatigue, for instance whether the perception is pleasant or unpleasant, and does not recognise different types of fatigue. While there may be situations where it would be helpful to understand the hedonic experience of fatigue perceptions, we concur with Enoka and colleagues [25] that distinguishing between types of fatigue through the use of accompanying descriptors such as central, mental and chronic is actually unnecessary, too vague to be useful and theoretically incoherent. The data from Experiments 3 and 4, to some extent, support this view because ROF outcomes, as a global quantitative measure of fatigue, were found to be a valid correlate of various associated constructs of fatigue as provoked in a variety of ways across different time-frames. For example, ROF was found to respond equally well to both short-duration exercise stimulants of fatigue seen in Experiment 3, and long-term daily activity stimulants of fatigue observed in Experiment 4. In essence, the ROF scale provides a means to measure fatigue as a singular perceptual phenomenon independent of hedonistic or typological variations.

Despite the encouraging results of Experiments 3 and 4, what we cannot conclude is whether times series measurements of perceived levels of fatigue can be reconciled in conditions that provoke both sudden and gradual changes in fatigue. For example, it is unclear what effect sudden episodes of fatiguing activity, perhaps of an unexpected or intermittent nature, would have on the daily time course of perceived fatigue or whether such changes can be adequately captured using the ROF scale. It is therefore a limitation of the present collection of experiments that the ROF has not been tested in this way over a continuous period of time incorporating both exercise and various daily living activities.

While we accept that, in many instances, covariance exists between fatigue and fatigability, if the predictions of Enoka and colleagues [2, 25] regarding interdependence are correct, then a number of seemingly counterintuitive situations could occur. For example, participating in exercise at the end of a working day might have an invigorating rather than fatiguing effect, despite exertion-related increases in physiological variables like heart rate, oxygen uptake and core temperature. In contrast, tasks high in cognitive effort but low in physical exertion could lead to significant ROF increases, similar to the effects demonstrated by Marcora and colleagues [11]. Because the ROF scale is not exercise- or context-specific it provides an opportunity to investigate these, and other, longitudinal changes in perceived fatigue across different time frames with combinations of activities.

### Future Rating-of-Fatigue Scale Development and Testing

In the preceding discussion, we have highlighted a variety of potential applications and experimental domains where the ROF scale has heuristic potential. While it is beyond the scope of this paper to exhaustively list ways in which the ROF might be developed and tested in the future, we are able to set out a selection of research questions that we consider to be important. The first is a measurement issue about the extent to which the ROF scale has utility in furthering our understanding of fatigue in terms of both the development of theory, and in understanding situational manifestations of fatigue symptoms. From an epidemiological perspective, a significant issue yet to be resolved is that no gold standard measure of fatigue exists [50]. As previously discussed, most fatigue scales were developed with specific conditions or populations in mind. Other novel attempts have created an energy index by combining the fatigue and vigour subscale scores of the Profile of Mood States [51], which is useful in some situations but not those where having to respond to a multiple item survey is prohibitive.

A second important area of study is to more clearly differentiate fatigue and exertion during physical activity. This is because most traditional exercise protocols create ideal conditions for construct mimicking, apparent from the high correlations reported in Experiment 3. A new experimental lens is needed to expose these mimicking effects and betray the difference between fatigue and exertion during exercise. A possibility would be to conduct a reverse ramp protocol in which we anticipate the high initial work load would produce high perceived exertion and low fatigue but as workload decreases increasing fatigue with decreasing perceived exertion. An alternative would be to examine the effect of exercise on RPE and ROF upon individuals in a sleep-deprived state, thus beginning with high ROF and low RPE which as exercise proceeds would see expected increases in RPE but we hypothesise reducing eves of fatigue.

The third question, related to the previous point, is what role exercise and other forms of physical activity have in moderating the symptoms of fatigue. A quantitative synthesis of relevant research found that chronic exercise provokes increased levels of energy [52] and a reduction in the risk of experiencing fatigue could be as high as 40% [51]. It is important to note that the effectiveness of various strategies and interventions on fatigue reduction is highly dependent on the validity of the particular method used to measure fatigue. In this regard, we believe the ROF scale could be a powerful instrument given that it can be quickly applied in just about any situation and thus will make longitudinal and cross-sectional comparisons easier.

## Conclusions

A new method of measuring fatigue incorporating an 11-point numerical scale with empirically derived accompanying descriptor and diagrammatic components has been developed and named the rating-of-fatigue (ROF) scale. In the series of experiments presented in this paper, the ROF scale was found to have good face validity and high levels of convergent validity during ramped cycling to exhaustion, resting recovery and daily living activities. Fatigue ratings also diverge from perceived exertion during recovery, highlighting the discriminant validity of the ROF scale. The ROF scale is an instrument that can track perceptions of fatigue across any range of living, physical activity or recovery contexts. The intensity-based approach to measuring fatigue adopted with the ROF scale, and the findings presented in the third and fourth experiments, appear to support theoretical notions that fatigue should be regarded as a global rather than context-specific perceptual phenomena.

### Electronic supplementary material

Below is the link to the electronic supplementary material.

Supplementary material 1 (DOCX 185 kb)

## Appendix A: The Rating-of-Fatigue Scale and Instructions

The rating-of-fatigue (ROF) scale will allow you to rate how fatigued you feel. The scale might be presented to you by another person or, in some circumstances, you might be asked to self-administer the scale. Whatever method is used, it is important that you first read the following guidelines:

1. Please familiarize yourself with the scale by looking closely at the ROF scale now. You will notice that the ROF scale consists of 11 numerical points that range from 0 to 10. There are also five descriptors and five diagrams that are intended to help you understand the scale and make you rating.
2. When you are presented with the ROF scale please carefully inspect the scale before giving a numerical response from 0 to 10. Always try to respond as honestly as possible giving a rating that best reflects how fatigued you feel at the time.
3. Try not to hesitate too much and make sure you only give ONE number as a response. For example, avoid responding by giving two numbers such as ‘three or four’.
4. Now please read the following examples of what some of the ROF ratings mean:

A response of 0 would indicate that you do not feel at all fatigued. An example of this might be soon after you wake up in the morning after having a good night’s sleep. Now try to think of a similar occasion in your past where you have experienced the lowest feelings of fatigue and use this as you reference.

A response of 10 would indicate that you feel totally fatigued and exhausted. An example of this might be not being able to stay awake, perhaps late at night but equally could include situations such as sprinting until you can no longer physically continue. Again try to think of a similar example that you have actually experienced in the past.
