# Supplementary figures and images for: Development and Validity of the Rating-of-Fatigue Scale
Source: Sports Med. 2017 Mar 10;47(11):2375–93. doi: 10.1007/s40279-017-0711-5 (PMC5633636; doi:10.1007/s40279-017-0711-5)

**
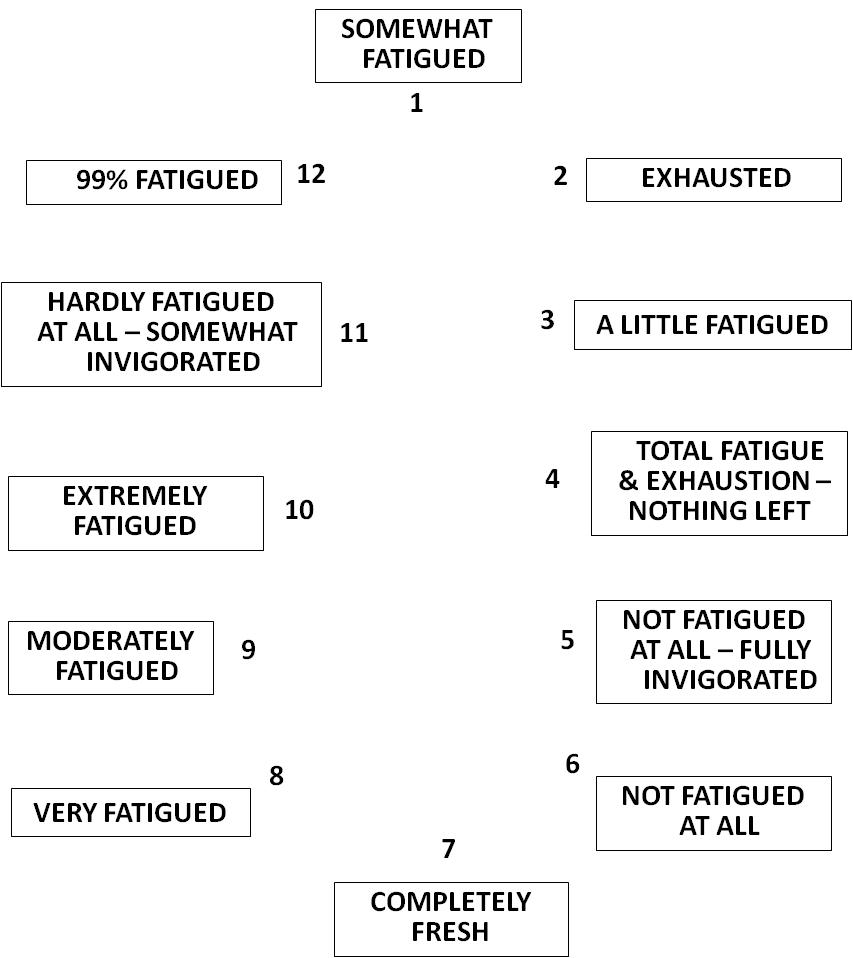
**

**
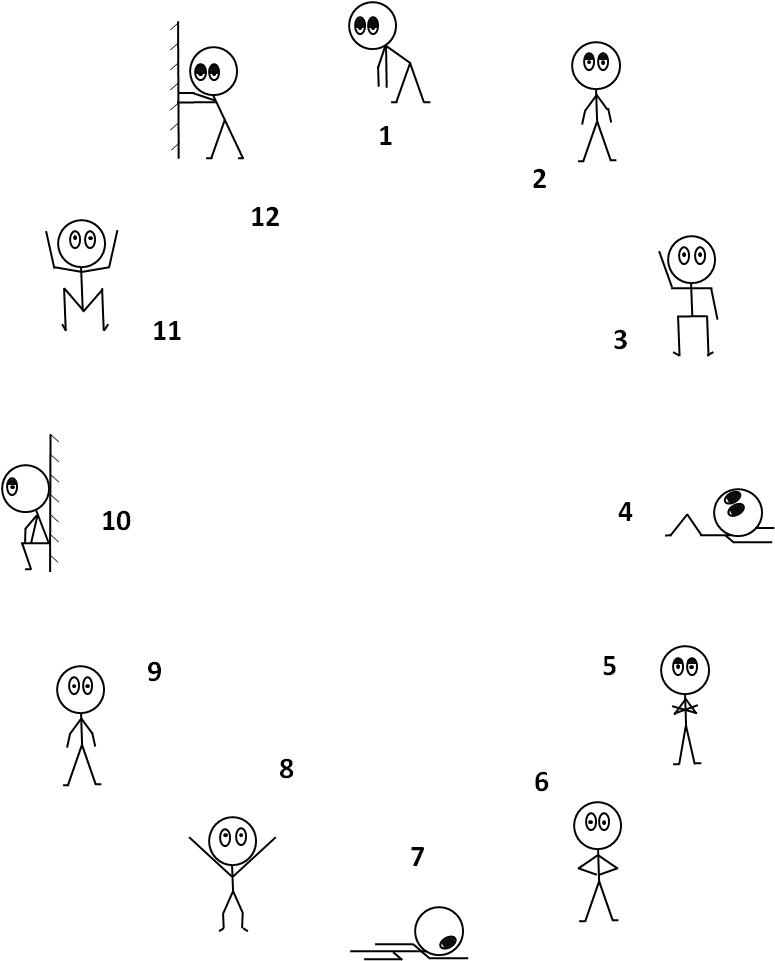
**

Supplement: Supplementary file 1 — Supplementary material 1 (DOCX 185 kb) [file 40279_2017_711_MOESM1_ESM.docx]
